# Unusual C=C Bond Isomerization of an α,β-Unsaturated γ-Butyrolactone Catalysed by Flavoproteins from the Old Yellow Enzyme Family

**DOI:** 10.1002/cbic.201200475

**Published:** 2012-09-28

**Authors:** Katharina Durchschein, Silvia Wallner, Peter Macheroux, Klaus Zangger, Walter M F Fabian, Kurt Faber

**Affiliations:** [a] Organic & Bioorganic Chemistry, Department of Chemistry, University of GrazHeinrichstrasse 28, 8010 Graz (Austria); [b] Institute of Biochemistry, Graz University of TechnologyPetersgasse 12, 8010 Graz (Austria)

**Keywords:** biocatalysis, C=C isomerization, flavins, isomerases, oxidoreductases

## Abstract

An unexpected, redox-neutral C=C bond isomerization of a γ-butyrolactone bearing an *exo*-methylene unit to the thermodynamically more favoured *endo* isomer (*k*_cat_ = 0.076 s^−1^) catalysed by flavoproteins from the Old Yellow Enzyme family was discovered. Theoretical calculations and kinetic data support a mechanism through which the isomerization proceeds through FMN-mediated hydride addition onto *exo*-Cβ, followed by hydride abstraction from *endo*-Cβ′, which is in line with the well-established C=C bond bioreduction of OYEs. This new isomerase activity enriches the catalytic versatility of ene-reductases.

## Introduction

α-Chiral lactones are important components for flavour and fragrance composites,[1, 2] such as jasmine,[3] mint,[4] whiskey,[5, 6] cognac[7–9] and wine lactone,[10–12] and are widely used as chiral building blocks for natural product synthesis.[2] One strategy to obtain them consists of the asymmetric bioreduction of the corresponding α,β-unsaturated precursors. Such “electronically activated” C=C bonds can be reduced by flavoproteins from the Old Yellow Enzyme (OYE) family, often in a stereocomplementary fashion.[13] Although this method has been successfully applied to a broad range of enals, enones, nitroalkenes and α,β-unsaturated carboxylic acids and their derivatives, such as esters or cyclic imides, unsaturated lactones had been only sparsely investigated.[14] In one example, β-substituted furan-2(5*H*)-ones were reduced by baker's yeast to give (*R*)-γ-butyrolactones with high stereoselectivity.[14, 15] In a related fashion, an α-substituted analogue was stereoselectively transformed by an NADH-dependent ene-reductase from the plant *Marchantia polymorpha* (liverwort).[15]

**Scheme 1:**
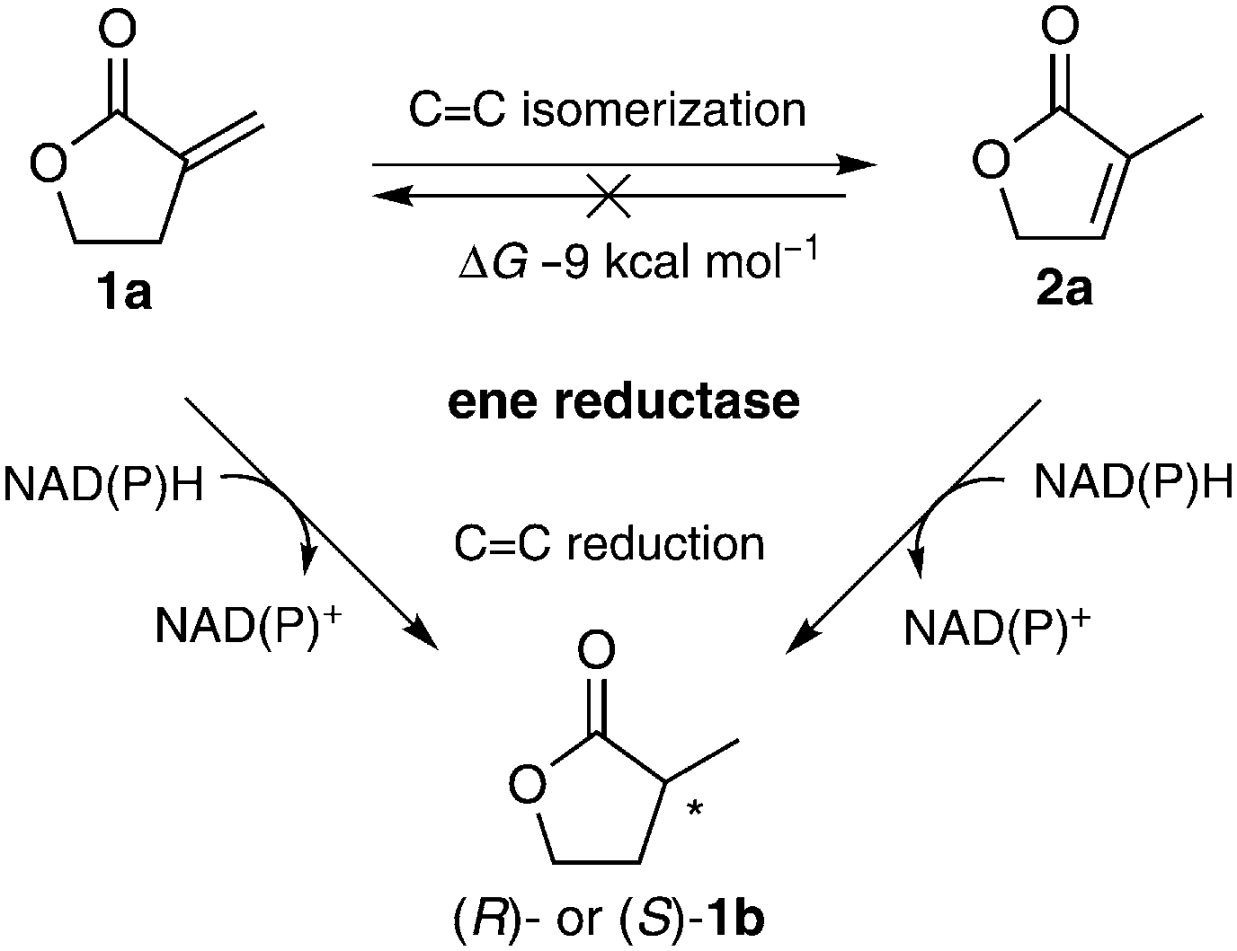
Biocatalytic C=C bond reduction and isomerization of α,β-unsaturated γ-butyrolactones **1 a** and **2 a**.

Even less is known about α,β-unsaturated δ-lactones: although baker's yeast was able to accept them as substrates, the stereorecognition of a chiral centre in the δ-position was very low.[16] In order to explore this substrate class for OYEs, we studied the bioreduction of two regioisomeric α,β-unsaturated γ-butyrolactones—α-methylene-γ-butyrolactone (**1 a**) and 3-methylfuran-2(5*H*)-one (**2 a**; Scheme 1)—through the action of a set of flavoproteins from the OYE family in the presence of NAD(P)H as hydride donor. Because the two regioisomeric substrates **1 a** and **2 a** should yield the same reduction product **1 b**, we anticipated potential stereocontrol, as previously shown for butenedioic, acrylic or cinnamic acid and their esters (Scheme 1).[17]

## Results and Discussion

Much to our surprise, the bioreduction of **1 a** furnished only minor amounts of the expected saturated lactone **1 b** with the regioisomer of the starting material (**2 a**) as main product (Scheme 1, Table 1). In contrast with the well-established C=C bond *reduction*,[13] redox-neutral C=C bond *isomerization* is an only rarely observed catalytic activity of flavoproteins.

**Table 1:** C=C bond bioreduction and isomerization of α,β-unsaturated γ-butyrolactones by ene-reductases

Substrate	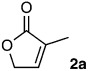			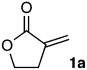		
Products	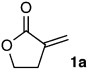	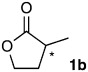		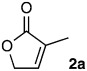	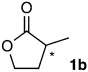	
Enzymes	Conv. [%]	Conv. [%]	*ee* [%]	Conv. [%]	Conv. [%]	*ee* [%]
EBP1	<1	75	20 (*R*)	78	20	49 (*R*)
OYE2	<1	16	48 (*R*)	76	24	75 (*R*)
OYE1	<1	17	35 (*R*)	70	25	84 (*R*)
KYE	<1	20	46 (*R*)	66	27	87 (*R*)
NCR	<1	13	11 (*R*)	11	27	85 (*R*)
XenA	<1	12	93 (*R*)	11	3	99 (*R*)
OYE3	<1	4	87 (*R*)	13	4	50 (*S*)
XenB	<1	2	93 (*R*)	<1	2	43 (*S*)
YqjM	<1	7	>99 (*R*)	<1	<1	n.d.
OPR1	<1	2	>99 (*R*)	<1	<1	n.d.
YhdA	<1	9	>99 (*R*)	<1	<1	n.d.
YcnD	<1	2	n.d.	<1	55	79 (*R*)

Reaction conditions: substrate (10 mm), Tris**⋅**HCl buffer (50 mm, pH 7.5), ene-reductase (protein concentration 100 μg mL^−1^), NADH (15 mm), 24 h, 30 °C, 120 rpm. n.d.: not determined, due to low conversion.

Reduced flavin is involved in a few non-redox rearrangement or isomerization reactions in the biosynthesis of isoprenoids (terpenes, carotenoids, sterols, ubiquinones and dolichols).[18, 19] The corresponding enzymes belong to the new class of FAD_red_-[20, 21] or FMN_red_-dependent non-redox enzymes,[22] such as isopentenyl-diphosphate isomerases type 2 (IDIs-2),[22] which catalyse the reversible isomerization of isopentenyl diphosphate (IPP) to dimethylallyl diphosphate (DMAPP), key precursors of isoprenoids.[19, 23, 24] *cis*–*trans* Isomerases,[20] such as lycopene-β-cyclase,[21] catalyse *E/Z* isomerizations of alkenes and are involved in the biosynthesis of β-carotenoids.

**Scheme 2:**
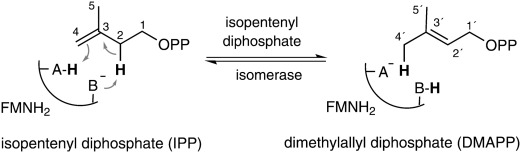
Proposed mechanism of C=C bond isomerization from isopentenyl diphosphate (IPP) to dimethylallyl diphosphate (DMAPP) catalysed by isopentenyl diphosphate isomerases type 2 (IDIs-2).[19, 34] B = N5 of FMNH_2_, A = N5-H of FMNH_2_ or N5-H_2_^+^ of protonated flavin, PP = diphosphate.

Interestingly, in all C=C-isomerization reactions the flavin cofactor must be in its reduced form,[25] although it does not change its redox state during catalysis.[26] So far, the exact role of reduced flavin during catalysis of redox-neutral C=C isomerizations is still a matter of discussion; the most recent mechanistic proposal involves a protonation/deprotonation sequence.[18, 27, 28] Of the FAD_red_/FMN_red_-dependent enzymes, IDIs-2 have been investigated in most detail (Scheme 2).[23, 29–33] During the isomerization of IPP to DMAPP, N5 of FMNH_2_ participates either directly (N5-H) or through a zwitterionic tautomer (N5-H_2_^+^) in mediating an allylic 1,3-proton addition/elimination at C2/C4 of IPP or C2'/C4' of DMAPP,[19, 34] in what does not seem to be a concerted mechanism.[35]

In all-*trans*-α,β-carotene biosynthesis, two of the final steps are isomerization/cyclization reactions. Carotene *cis-trans* isomerase (CrtIso from tomato), for instance, catalyses the *cis-trans* isomerization of prolycopene (7,9,9′,7′-tetra-*cis*-lycopene) to all-*trans*-lycopene, which is subsequently (redox-neutral) cyclized by lycopene cyclase CrtY to form all-*trans*-α,β-carotenes (Scheme S1 in the Supporting Information). CrtIso shows strong mechanistic similarities with IDIs-2, although its mechanism has been investigated to a lesser extent. Again, N5 of FADH_2_ was suspected to act as an acid/base catalyst, similarly to IDIs-2.[20] In contrast, CrtY—which cyclizes the linear C_40_-carotene lycopene to provitamin A carotenoids[21]—is active with C5-deaza-FADH_2_, unlike IDIs-2 and CrtIso, for which N5 of FMNH_2_ is required for activity.[20] It was thus proposed that anionic FADH_2_ (N5) of CrtY stabilizes the cationic intermediate.[21]

Unlike in the case of substrate **1 a**, a variety of ene-reductases were able to reduce isomer **2 a** at the expense of NADH by forming the expected saturated lactone **1 b** in up to 75 % yield (Table 1, left). Whereas the most active enzyme (EBP1) showed only low stereoselectivity (20 % *ee*), the “slow” proteins YqjM, OPR1 and YhdA gave (*R*)-**1 b** in up to >99 % *ee*.[36] No switch in stereoselectivity could be detected, neither could any trace of C=C-isomerization product **1 a**.

A completely different picture was seen with the regioisomeric α-methylene lactone **1 a** (Table 1, right). Although this substrate was transformed more rapidly (93–100 % total conversion) by several enzymes (EBP1, OYE1, OYE2, KYE), the expected reduction product **1 b** was formed in only minor amounts (up to 27 %), whereas the major product turned out to be isomer **2 a** (66–78 % conversion). Whereas NCR, XenA, OYE3 and XenB showed low activities, YcnD gave only reduction product (*R*)-**1 b**, with 55 % conversion and in 79 % *ee*. In addition, the stereoselectivities could be switched between (*R*)-**1 b** (*ee* up to 99 %, XenA) and (*S*)-**1 b** (*ee* up to 50 %, OYE3).

To obtain insight into the mechanism of this unexpected biocatalytic C=C-isomerization, we investigated the kinetics and the influence of reaction conditions in more detail, using OYE2 as test protein. As depicted in Scheme 1, the overall system consists of two competing pathways: direct bioreduction of **1 a** to yield **1 b**, or isomerization of **1 a** to **2 a**, which is subsequently reduced to **1 b**. Fortunately, all three individual reactions can be considered irreversible, because the reduction of conjugated C=C bonds at the expense of nicotinamide is strongly favoured, due to the fact that two very strong C–H bonds are being formed at the expense of a (weaker) π bond. Consequently, the reverse (desaturation) reaction catalysed by an exceptionally thermostable OYE from *Geobacillus kaustophilus* required elevated temperatures of ca. 70 °C.[37] The isomerization of the *exo*-methylene moiety in **1 a** to form isomer **2 a** is equivalent to the conversion of a (less favoured) terminal alkene to a (more stable) internal one. Coupled cluster calculations gave a Δ*G* value of ≍−9 kcal mol^−1^. In addition, **1 a** and **2 a** contain *s-cis* and *s-trans* heterodiene subunits, respectively. The *s-trans* conformer of 2-methylacrolein was calculated [CCSD(T)] to be more stable by 3.7 kcal mol^−1^ (see the Supporting Information).

**Figure 1:**
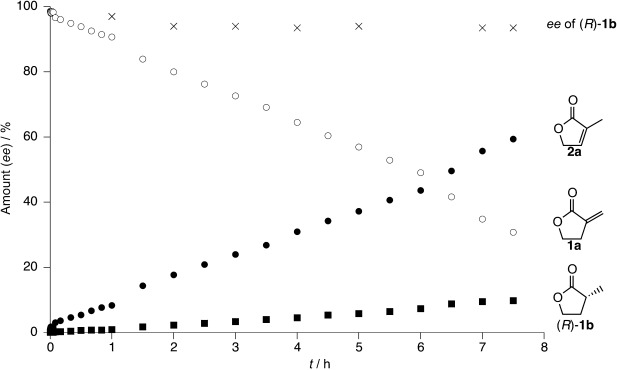
Time courses of C=C isomerization and of reduction of α-methylene-γ-butyrolactone (**1 a**), both catalysed by OYE2. Substrate **1 a**: ○. Isomerization product **2 a**: •. Reduction product (*R*)-**1 b**: ▪ (conversion) and × (*ee*). Final end-point data after 26 h were 78 % **2 a** and 22 % (*R*)-**1 b** (78 % *ee*). Reaction conditions: substrate (10 mm), ene-reductase OYE2 (100 μg mL^−1^), NADH (15 mm), Tris**⋅**HCl buffer (50 mm, pH 7.5), 30 °C, 120 rpm.

In order to elucidate the stereoselectivities of the bioreductions of the regioisomeric alkenes **1 a** and **2 a**, time studies were performed. The reduction of **2 a**, which is not impeded by competing isomerization, produced (*R*)-**1 b** with a modest *ee* of 48 % after 24 h (Table 1, left column, entry 2). In contrast, with **1 a** as substrate, the *ee* of (*R*)-**1 b** was shown to be >99 % at the onset of the reaction, when the concentration of **2 a** was a minimum, and it gradually declined to 75 % *ee* over 26 h (Figure 1). This is due to the low stereoselectivity of the bioreduction of **2 a**, which is gradually formed by isomerization. Generally, the isomerization (78 % conversion) was three times faster than C=C reduction (22 % conversion) over 26 h. Detailed measurements revealed OYE2 to have *k*_cat_ values of 0.076 s^−1^ for isomerization and 0.013 s^−1^ for reduction, the latter being five orders of magnitude lower than the *k*_cat_ values of typical isomerases such as type II isopentenyl-diphosphate isomerases (IDIs-2) from *Thermus thermophilus* and from *Synechocystis* sp., which have *k*_cat_ values of 17.9×10^2^ and 23×10^2^ s^−1^, respectively.[24]

It is interesting to note that the isosteric α,β-unsaturated cyclopentene analogue of lactone **2 a** (i.e., 2-methylcyclopent-2-en-1-one) was reduced by OYE2 without stereoselectivity;[38] this demonstrates that the additional oxygen atom in lactone **2 a** significantly enhanced the chiral recognition.

It is known that the natures of the nicotinamide cofactors can have implications for the catalytic performances of OYEs,[39] so the following experiments were performed with OYE2 and substrate **1 a** (Table 2). No reaction occurred in the absence of enzyme or with (oxidized or reduced) nicotinamide alone (entries 1–4). Interestingly, OYE2 strictly required the reduced cofactor for activity (entries 5–7); this parallels the activities of isomerases discussed above.[25] A preference for NADH over NADPH was clearly visible (entries 6 and 8). Finally, analogous control experiments were performed by replacing OYE2 with albumin to ensure that the catalytic activity of OYE2 can be attributed to its active site and not to (unspecific) catalysis by polar amino acid residues at the protein surface (entries 9–11). Furthermore, no trace of desaturation could be detected when **1 b** was used as substrate under the above conditions.

**Table 2:** Influence of reaction conditions on C=C isomerization and reduction of substrate 1 a

		Substrate	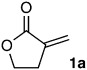	
		Products	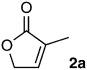	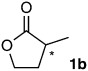
	Protein	Cofactor	Conv. [%]	Conv. [%]
1	OYE2	none	<1	<1
2	none	NADH	<1	<1
3	none	NAD^+^	<1	<1
4	none	NAD^+^/NADH (1:1)	<1	<1
5	OYE2	NAD^+^	<1	<1
6	OYE2	NADH	74	26
7	OYE2	NAD^+^/NADH (1:1)	78	21
8	OYE2	NADPH	30	2
9	albumin	none	<1	<1
10	albumin	NADH	<1	<1
11	albumin	NAD^+^	<1	<1

Reaction conditions: substrate (10 mm), protein (OYE2, concentration 100 μg mL^−1^), albumin (≍1 mg), cofactor (15 mm), Tris**⋅**HCl buffer (50 mm, pH 7.5), 24 h, 30 °C, 120 rpm.

### Kinetic studies

In order to unravel the network of competing C=C reduction and isomerization reactions, the kinetic rates for the conversion of substrates **1 a** and **2 a** were determined. Different substrate concentrations (0.1–4 mm) were used to follow conversion as a function of time, which allowed us to calculate the individual catalytic rates of isomerization and reduction reactions. From the data in Table 3 it can be deduced that:

**Table 3:** Kinetic constants for C=C bond reduction and isomerization of regioisomeric substrates 1 a and 2 a in the presence of OYE2 (average values of triplicate measurements)[a]

Substrate	Reduction			Isomerization		
	*K*_M_ [mm]	*k*_cat_ [s^−1^]	*k*_cat_/*K*_M_ [s^−1^ mm^−1^]	*K*_M_ [mm]	*k*_cat_ [s^−1^]	*k*_cat_/*K*_M_ [s^−1^ mm^−1^]
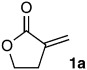	0.76±0.09	0.015±0.0005	1.97×10^−8^	1.62±0.28	0.10±0.01	6.18×10^−8^
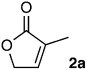	1.12±0.06	0.0056±0.0003	0.50×10^−8^	n.r.	n.r.	n.r.

[a] Reaction conditions: substrate (0.1–4 mm), Tris**⋅**HCl buffer (50 mm, pH 7.5), NADH (15 mm), 2 h, 30 °C. n.r. no reaction.

1. The *K*_M_ values for regioisomeric lactones **1 a** and **2 a** are of the same order of magnitude for all three reactions, with (760±90) μm (**1 a**) and (1120±160) μm (**2 a**) for reduction, whereas the *K*_M_ of **1 a** for isomerization is slightly higher at (1620±280) μm. These rather high *K*_M_ values indicate that none of the compounds is a perfect substrate for OYE2. In contrast, the *K*_M_ values for IDIs-2 from *T. thermophilus* and from *Synechocystis* sp. are three or two orders of magnitude lower (5.6 and 52 μm, respectively), which indicates tighter binding.[24]
2. In contrast, significant differences exist in the catalytic rates. Overall, C=C isomerization of **1 a** to **2 a** is the fastest reaction (*k*_cat_ = 0.10±0.01 s^−1^), whereas the subsequent reduction of **2 a** to **1 b** is 18 times slower (0.0056±0.0003 s^−1^). The reduction of the thermodynamically more stable isomer **2 a**, which cannot be isomerized, is about three times slower than the direct reduction of **1 a** to **1 b**.

The kinetic data make it clear that C=C bond *isomerization* catalysed by OYE2—the main role of which is C=C bond *reduction*—constitutes a side activity, thereby reflecting the versatility of the flavin cofactor.[40]

For FAD_red_/FMN_red_-dependent isomerization reactions, equimolar amounts of cofactor and enzyme are enough to ensure continuous (redox-neutral) isomerization cycles.[26] OYE2 was therefore reduced with equal amounts of NADH (20 μm each) and the reduced enzyme was applied to convert various amounts of substrate **1 a** (0.5 mm–20 mm) under an oxygen-free atmosphere to prevent reoxidation of the reduced flavin by O_2_. In these experiments, the degrees of isomerization increased on going from 30 μm (0.5 mm substrate **1 a**) to 1400 μm (20 mm substrate **1 a**; see the Supporting Information). This is due to the prevention of O_2_ oxidation of FMNH_2_, which would produce catalytically inactive oxidized flavin.

### ^2^H-labelling experiments

In an attempt to elucidate the hydride migration pathway during C=C bond isomerization, experiments on the bioreduction/isomerization of substrates **1 a** and **2 a** were performed in the presence of deuterated nicotinamide (NADD) and flavin cofactors (FMND_2_), obtained with the aid of the [D_8_]propan-2-ol/NAD^+^/ADH-A recycling system[41] in D_2_O. Deuteration was monitored by ^13^C and ^2^H NMR spectroscopy (Table S3). Incorporation of deuterium leads to the appearance of triplets with equal line intensities in the ^13^C spectra, due to the coupling with the spin 1 nucleus ^2^H. With α-methylene-γ-butyrolactone (**1 a**) as substrate, the major product was non-deuterated 3-methyl-furan-2(5*H*)-one (**2 a**) together with a minor amount of *mono*-deuterated 3-[D]methyl-furan-2(5*H*)-one (**2 a**) in a ratio of ≍3:1; this suggests internal hydride [H^−^] transfer from the *endo*-β′-position of the enolate intermediate to the *exo*-β-position through the FMN cofactor. The minor amounts of *mono*-deuterated isomerization product arise from the onset of the reaction, when FMN is predominantly deuterated, and from (spontaneous) H/D exchange between reduced flavin and the solvent;[42] this excludes experiments based on FMNH_2_ in D_2_O and vice versa.

### Proposed C=C bond isomerization mechanism

**Scheme 3:**
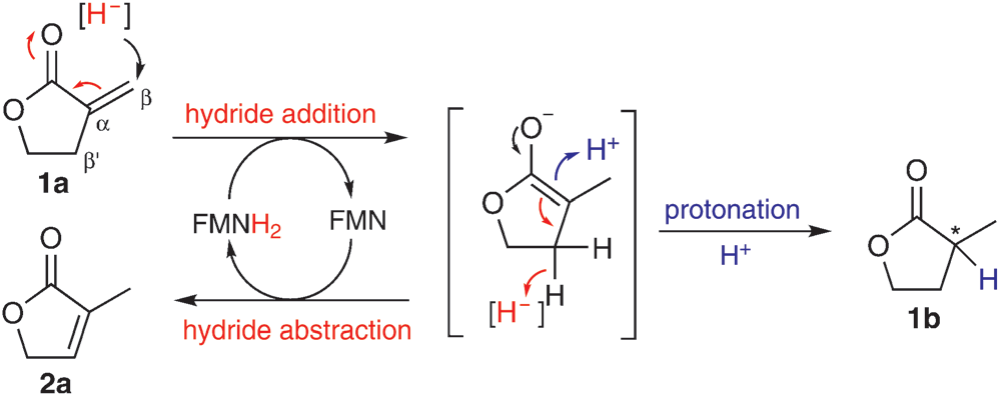
Proposed mechanism for C=C bond isomerization through hydride migration (red) versus C=C bond reduction (blue).

Based on the obtained data, a mechanism for the C=C bond isomerization is suggested (Scheme 3). To initiate isomerization and reduction, FMN needs to be reduced by NADH. Hydride attack from N5 of reduced flavin occurs onto Cβ of the *exo*-methylene moiety of substrate **1 a** to yield a resonance-stabilized enolate intermediate consistently with the mechanisms of ene-reductases,[43, 44] which resemble Michael-type nucleophilic 1,4-addition.[45] At this point, the reaction branches into two different pathways:

1. Protonation of the enolate (through a Tyr or Cys residue in the active site[46]) at Cα completes C=C bond reduction by forming the saturated dead-end product **1 b**. To start a consecutive reductive cycle, the oxidized flavin has to be reduced by another nicotinamide hydride.
2. Alternatively, the enolate is stabilized by hydride abstraction at the *endo*-Cβ′ position to produce the (thermodynamically favoured) isomer **2 a**, together with recycling of reduced flavin in a redox-neutral mode.

## Conclusions

An unusual redox-neutral C=C bond isomerization reaction of a γ-butyrolactone bearing an *exo*-methylene unit to produce the thermodynamically more favoured *endo* isomer, catalysed by flavoproteins from the Old Yellow Enzyme family, has been discovered. This adds a new facet to the unclear physiological function of these enzymes. Kinetic data suggest that the isomerization is initiated by hydride addition onto *exo*-Cβ to yield an enolate intermediate, which is stabilized by hydride abstraction from *endo*-Cβ′ mediated by the FMN cofactor. This isomerase-like activity enhances the catalytic versatility of OYE-flavoproteins and is in line with their well-established capacity for C=C bond bioreductions.[13]

## Experimental Section

**General**: NMR spectra were measured with Bruker Avance III 300 MHz and Bruker Avance III 700 MHz NMR spectrometers at 298 K. Deuterium NMR spectra were acquired with the 300 MHz spectrometer and a 5 mm BBO probe, at a ^2^H frequency of 46 MHz. Indirect field-frequency locking was achieved through pseudo-locking by acquiring series of short 1D spectra and shifting the transmitter after each spectrum as previously described.[47] ^13^C NMR spectra to pinpoint the deuteration site were obtained with the aid of inverse gated ^1^H decoupled spectra with the 700 MHz NMR and a 5 mm TCI cryo-probe. Proton coupling constants are given in Hz. Petroleum ether (b.p. 60–90 °C) and EtOAc used for chromatography were distilled prior to use.

**General procedures for biotransformations, time studies and recycling systems**: An aliquot of enzyme (OYE1–3, OPR1, YqjM, YhdA, NCR, XenA, XenB, TS, YcnD or EBP1, protein concentration 100 μg mL^−1^) was added to a Tris**⋅**HCl buffer solution (0.8 mL, 50 mm, pH 7.5) containing the substrate (10 mm) and the cofactor NADH (15 mm). The mixture was shaken at 30 °C and 120 rpm. After 24 h, products were extracted with EtOAc (2×0.5 mL). The combined organic phases were dried over Na_2_SO_4_ and analysed by achiral GC to determine the degree of conversion and by chiral GC or HPLC to determine the enantiomeric excess. For cofactor recycling, the oxidized form of the cofactor [NAD(P)^+^, 100 μm], the cosubstrate (glucose or glucose-6-phosphate, 20 mm) and the recycling enzyme (glucose dehydrogenase or glucose-6-phosphatedehydrogenase, 10 U) were used.

**Control experiments**: An aliquot of enzyme [OYE (100 μg mL^−1^) or albumin (1 mg)] was added to a Tris**⋅**HCl buffer solution (0.8 mL, 50 mm, pH 7.5), containing the substrate **1 a** or **1 b** (10 mm), either in the presence or in the absence of cofactor NADH (15 mm). The mixture was shaken at 30 °C and 120 rpm. After 24 h products were extracted with EtOAc (2×0.5 mL). The combined organic phases were dried over Na_2_SO_4_ and analysed by achiral GC.

**Kinetic measurements**: *K*_M_ and *k*_cat_ values for the isomerization and reduction of substrates **1 a** and **2 a** were calculated from Michaelis–Menten plots. Initial velocities for substrate turnover were determined by use of various substrate concentrations (0.1–4 mm) in reaction mixtures (0.8 mL) each containing an aliquot of enzyme (OYE2, final protein concentration 100 μg mL^−1^) in Tris**⋅**HCl buffer solution (50 mm, pH 7.5) with NADH (15 mm). The mixtures were incubated at 30 °C and 120 rpm. After 2 h, substrate turnover was stopped by addition of EtOAc (500 μL) followed by vortexing. The combined organic phases were dried over Na_2_SO_4_ and analysed by achiral GC to determine the degrees of conversion. Triplicate experiments were performed. Initial velocities for substrate turnover were calculated and plotted against the corresponding substrate concentrations, and *K*_M_ and *k*_cat_ values were determined by nonlinear regression in Origin 7.0.

Studies to determine the catalytic rates for isomerization and reduction (*k*_cat_) were also performed. A typical assay consisted of Tris**⋅**HCl (0.8 mL, 50 mm, pH 7.5), an aliquot of enzyme (OYE2, final protein concentration 100 μg mL^−1^), NADH (15 mm) and substrate **1 a** or **1 b** (0.1–4 mm) at 30 °C and 120 rpm. Reactions were stopped at different time points (90 min to 450 min) by product extraction with EtOAc as described above. Product formation was monitored by achiral GC.

**Determination of absolute configuration**: The absolute configuration of enantioenriched α-methyl-γ-butyrolactone (**1 b**) was determined by measurement of its optical rotation after isolation of the product from an up-scaled bioreduction of **1 a** in the presence of EBP1 from *Candida albicans*. Thus, 60 samples, each containing **1 a** (10 mm), NAD(P)H (15 mm) and EBP1 (200 μg mL^−1^) in a total volume of 800 μL, were shaken for 48 h at 30 °C and 120 rpm. The product was extracted three times with ethyl acetate, and the solvent was evaporated to yield **1 b** (9.4 mg). The optical rotation value [*α*]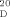 was measured with a PerkinElmer polarimeter 341 at 589 nm (Na D line) in a 1 dm cuvette: [*α*]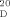 = +12.6 (*c* = 0.72, EtOH), *ee* ((*R*)-**1 b**) = 49 % [Lit. (*R*)-**1 b**: [*α*]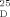 = +24.9 (*c* = 0.34, CHCl_3_);[15, 48] (*S*)-**16 b**: [*α*]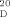 = −22.9 (*c* = 2, EtOH);[49] [*α*]_D_ = −21.5 (*c* = 5.5, EtOH)[50]]. Additional verification was achieved by comparison of the elution order on chiral HPLC with literature data.[51]

## Supplementary material

Detailed facts of importance to specialist readers are published as ”Supporting Information”. Such documents are peer-reviewed, but not copy-edited or typeset. They are made available as submitted by the authors.
