# Supplementary material for: Unusual C=C Bond Isomerization of an α,β-Unsaturated γ-Butyrolactone Catalysed by Flavoproteins from the Old Yellow Enzyme Family
Source: Chembiochem. 2012 Sep 28;13(16):2346–51. doi: 10.1002/cbic.201200475 (PMC3533789; doi:10.1002/cbic.201200475)
Supplement: Supplementary file 1 [file cbic0013-2346-SD1.pdf]

CHEMBIOCHEM

Supporting Information

© Copyright Wiley-VCH Verlag GmbH & Co. KGaA, 69451 Weinheim, 2012

Unusual C=C Bond Isomerization of an α,β-Unsaturated γ-Butyrolactone Catalysed by Flavoproteins from the Old Yellow Enzyme Family

Katharina Durchschein,[a] Silvia Wallner,[b] Peter Macheroux,[b] Klaus Zangger,[a]
Walter M. F. Fabian,[a] and Kurt Faber*[a]

cbic_201200475_sm_miscellaneous_information.pdf

Electronic Supporting Information

C=C-Bond isomerization/cyclization in β-carotene biosynthesis	S2
Source of chemicals, cofactors and enzymes	S2
Theoretical calculations	S3
Isomerization under inert atmosphere	S3
Deuterium labelling studies	S4
Analytical methods	S5
References	S6

Scheme S1. C=C-Bond isomerization/cyclization in β-carotene biosynthesis catalyzed by carotene *cis-trans* isomerase (CrtIso) and lycopene cyclase (CrtY).

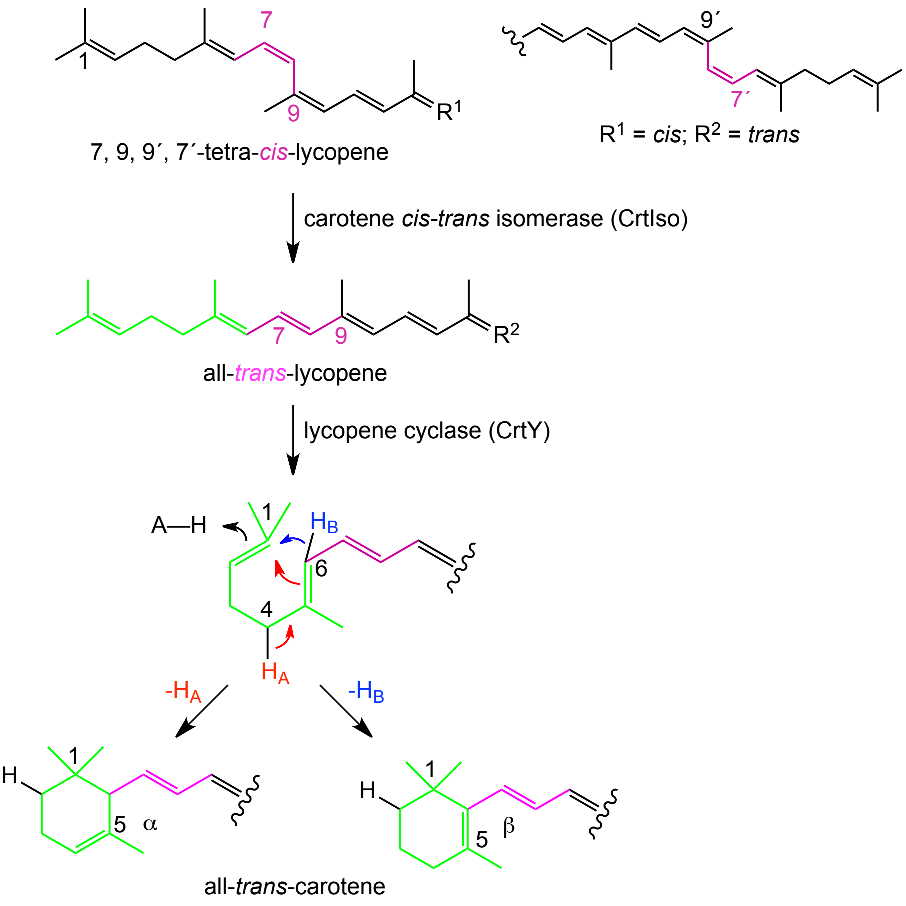

Source of chemicals, cofactors and enzymes

α-Methylene-γ-butyrolactone (**1a**), 3-methyl-furan2(5H)-one (**2a**), *rac*-α-methyl-γ-butyrolactone (**1b**) and bovine albumin were purchased from Sigma Aldrich, glucose and NAD^+ were from Fluka. NADH and NADPH were from AppliChem and $NADP^+$ was from Biocatalytics. Glucose-6-phosphate and glucose-6-phosphate dehydrogenase were obtained from Biochemica and glucose dehydrogenase were obtained from Jülich Chiral Solutions. *i*-Propanol-d_8 (label >99%) was from Sigma Aldrich.

Preparation of enzymes

OPR1 from *Lycopersicon esculentum*, YqjM[1,2,3] and YhdA[4] from *Bacillus subtilis* were overexpressed and purified as previously reported. Cloning and purification of OYEs from yeasts (OYE1 from *Saccharomyces carlsbergensis*, OYE2 and OYE3 from *Saccharomyces cerevisiae*) and nicotinamide-dependent cyclohexenone reductase (NCR) from *Zymomonas mobilis* was performed according to literature.[5,6] Xenobiotic reductases A (XenA) and B (XenB) from *Pseudomonas putida* and *P. fluorescens*, respectively,[7] and estrogen-binding protein (EBP1) from

Candida albicans were obtained as recently published.[8] KYE was provided by A. Bommarius (Georgia Institute of Technology, Atlanta, USA), the expression and purification was followed according to the published protocol[9] with an additional gel filtration step using Superdex 75. Ycnd was cloned, expressed and purified according to the literature.[10]

Theoretical calculations

Geometries were optimized by second order perturbation theory[11] using Dunning's double-zeta correlation consistent basis set[12] (MP2/cc-pVDZ). Zero point energy and thermal corrections to Gibbs free energies were obtained by the standard harmonic oscillator-rigid rotor approximation using the same level of theory. The complete basis set limit (MP2/CBS) was estimated using Martin's extrapolation scheme[13] in combination with the cc-pVnZ, n = 2-4, basis set. Higher electron correlation effects were estimated by coupled-cluster calculations[14] in combination with a two-point extrapolation[15] to the basis set limit [CCSD(T)/cc-pVnZ, n = 2-3]. Solvent effects (aqueous solution) were estimated by the SM8 solvation model[16] using the M06-2X density functional[17] in combination with the 6-31+G(d,p) basis set.[18] Programs used were: NWChem 6.0,[19] GAMESSPLUS,[20] and MOLDEN[21] for visualization. The ΔG of the isomerization reaction of **1a** to **2a** was determined to be -9 kcal/mol.

Table S1. Gibbs free energies [kcal mol^{-1}] of isomerization of **1a** to **2a**.

Method	Gas phase	Aqueous solution
MP2/CBS	8.7	9.5
CCSD(T)/CBS	8.0	8.8

Isomerization under inert atmosphere

An aliquot of enzyme (OYE2, final protein concentration 10 μM) in Tris-HCl buffer solution (50 mM, pH 7.5) was mixed with equal amounts of NADH (10 μM) to fully reduce the flavin cofactor. The reduced enzyme solution was then supplemented with substrate **1a** at various concentrations (0.1 mM - 20 mM) and reaction mixtures were incubated at 30 °C and 120 rpm in an oxygen-free atmosphere in a glove box (Belle Technology, ~0.8 ppm O_2). After 24 h the reactions were stopped and the product was extracted with EtOAc (2 × 0.5 mL). The combined organic phases were dried over Na_2SO_4 and analysed on achiral GC to determine the conversion.

Table S2. Isomerization of **1a** to **2a** using equimolar amounts of NADH (10 μM) and OYE2 (10 μM) under exclusion of O_2.

**Substrate [mM]** 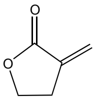 **1a**	**Product [μM]** 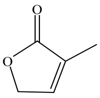 **2a**
0.5	30
1	30
5	50
10	400
20	1400

Reaction conditions: NADH (10 μM), OYE2 (10 μM), **1a** (0.5 - 20 mM), 24 h, N_2 atmosphere; n.d. = not determined;

Deuterium labeling studies

For up-scaling of the biotransformation of **1a**, 60 samples were prepared in the following way: an aliquot of enzyme (OYE2 200 *μ*g/mL) was added to a Tris-HCl D_2O buffer solution (0.8 mL, 50 mM, pH 7.5) containing the substrate (10 mM), the cofactor NAD^+ (100 *μ*M), the recycling-system ADH-A (10 U), *i*-propanol-d_8 (15 mM). The mixture was shaken at 30 °C and 120 rpm. After 48h the products were extracted with $CDCl_3$ (2 × 0.5 mL). The combined organic phases were dried over Na_2SO_4 and analysed via ^{13}C- and ^2H-NMR. For the acquisition of ^2H spectra $CDCl_3$ was evaporated and replaced by $CHCl_3$.

α-Methylene-γ-butyrolactone (**1a**): ^1H-NMR (300MHz, $CDCl_3$): δ 2.94-2.99 (2H, m), 4.36 (CH_2-O, t, J=7.35), 5.66 (1H, s), 6.23 (1H, s); ^{13}C-NMR (75MHz, $CDCl_3$): δ 27.38, 65.34, 122.3, 133.6, 170.8. NMR data corresponded to literature.[22, 23]

3-Methylfuran-2(5H)-one (**2a**): ^1H-NMR (300MHz, $CDCl_3$): δ 1.91 (3H, m), 4.74 (2H, m), 7.13 (1H, m); ^{13}C-NMR (75MHz, $CDCl_3$): δ 10.76, 70.12, 129.93, 145.11, 174.96. NMR data corresponded to literature.[24] For partially deuterated **2a**: ^{13}C-NMR (700MHz, $CDCl_3$): δ 10.70 (CH_3), 10.35, 10.46, 10.57 (CH_2-D), 70.02 (OCH_2), 129.92 (CH=C), 144.95 (C=CH), 174.96 (CO).

α-Methyl-γ-butyrolactone (**1b**): ^1H-NMR (300MHz, $CDCl_3$): δ 1.27 (3H, d, J=7.02), 1.87-1.97 (1H, m), 2.37-2.46 (1H, m), 2.55-2.63 (1H, m), 4.13-4.21 (1H, m), 4.29-4.36 (1H, m); ^{13}C-NMR (75MHz, $CDCl_3$): δ 15.28, 30.81, 34.26, 66.37, 180.3. NMR data corresponded to literature.[25] ^2H-NMR (46 MHz, $CHCl_3$): δ 2.66, 4.80.

Table S3. ^{2}H-Labeling experiments.

Conditions	Products (relative amounts)[a]
D_2O Tris-HCl 50mM pH 7.5/ OYE2/**1a**/NADH [b]	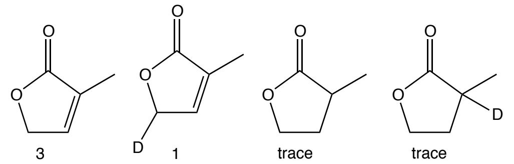
H_2O Tris-HCl 50mM pH 7.5/ OYE2/**1a**/*i*-PrOH-d_8/ADH-A/NAD^+ [c]	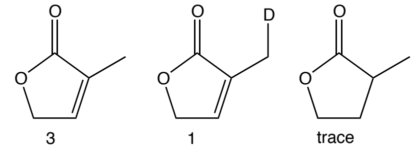
D_2O Tris-HCl 50mM pH 7.5/ OYE2/**1a**/*i*-PrOH-d_8/ADH-A/NAD^+ [d]	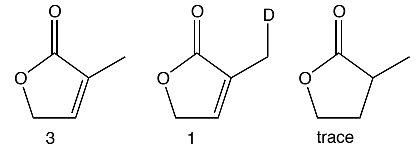

[a] Monitored via ^{13}C-NMR (700MHz spectrometer) and ^{2}H-NMR (300 MHz spectrometer); [b] H/D-scrambling of $FMNH_2$ with D_2O involves deuteration on C4 of **2a**; [c] H/D-scrambling between $FMND_2$ or FMNHD in H_2O is not visible; [d] no H/D-scrambling.

Analytical methods

GC-MS analyses were performed on a HP 6890 Series GC system equipped with a 5973 mass selective detector and a 7683 Series injector using a (5%-phenyl)-methylpolysiloxane capillary column (HP-5Msi, 30 m x 0.25 mm, 0.25 μm film). GC-FID analyses were carried out on a Varian 3800 using H_2 as carrier gas (14.5 psi). HPLC analyses were performed on a Shimadzu system equipped with a Chiralcel OD-H column (25 cm x 0.46 cm).

Determination of conversion

Table S3 Determination of conversion via achiral GC-analyses.

Substrate	Column	Program	Compound	T_{ret} [min]	Compound	T_{ret} [min]
**1a**	A	a	**1a**	9.23	**1b**	7.87
**2a**	A	a	**2a**	9.02	**1b**	7.87
**1a**	B	b	**1a**	7.08-7.09	**1b**	6.72-6.75
**2a**	B	b	**2a**	6.98-7.03	**1b**	6.72-6.75

Columns:

A = DB-1701 (14 % cyanopropyl-phenylphase capillary column, 30 m x 0.25 mm, 0.25 μm film), H_2 as carrier gas (14.5 psi), detector temperature 250 °C, split ratio 20:1.

B= GC-MS, HP-5 Msi (5%-phenyl-methylpolysiloxane capillary column, 30 m x 0.25 mm, 0.25 μm film), 0.69 psi H_2 as carrier gas, front inlet temperature 250 °C, split ratio 90:1.

Programs:

a = 110 °C, hold 5 min, 30 °C/min to 200 °C, hold 2 min.

b = 40 °C, hold 2 min, 20 °C/min to 180 °C, hold 1min.

Table S4. Determination of enantiomeric excess via chiral GC- and HPLC-analyses.

Substrate	Column	Conditions	Substrate (**1a**, **2a**)	Product **1b**	
			T_{ret} [min]	T_{ret} [min] (*R*)	T_{ret} [min] (*S*)
**1a**	C	c	n.d	**1b** 63.92	**1b** 69.31
	D	d	**1a** 20.4<br>**2a** 19.38	**1b** 15.40	**1b** 15.21
	E	d	**1a** 11.61<br>**2a** 11.44	**1b** 11.23	**1b** 10.82
**2a**	C	c	n.d	**1b** 63.92	**1b** 69.31
	D	d	**2a** 19.38	**1b** 15.40	**1b** 15.21
	E	d	**2a** 11.44	**1b** 11.23	**1b** 10.82

n.d. not determined.

Columns:

C = HPLC analysis employing a Chiralcel OD-H column (25 cm x 0.46 cm).

D = GC-analysis using a trifluoroacetyl β-cyclodextrin capillary column, detector temperature 200 °C, split ratio 20:1, H_2 as carrier gas (14.5 psi)

E = GC-analysis using a hydrodex-β-6TBDM capillary column, detector temperature 200 °C, split ratio 20:1, H_2 as carrier gas (14.5 psi)

Conditions:

c = *n*-heptane/2-propanol 99:1, 0.25 mL/min, detection at 216 nm.

d = 120 °C, hold 20 min, 15 °C/min to 180 °C, hold 2 min.

References

[1] C. Breithaupt, R. Kurzbauer, H. Lilie, A. Schaller, J. Strassner, R. Huber, P. Macheroux, T. Clausen, *Proc. Natl. Acad. Sci. USA* **2006**, *103*, 14337-14342.

[2] K. Kitzing, T. B. Fitzpatrick, C. Wilken, J. Sawa, G. P. Bourenkov, P. Macheroux, T. Clausen,

J. Biol. Chem. **2005**, *280*, 27904-27913.
[3] M. Hall, C. Stueckler, W. Kroutil, P. Macheroux, K. Faber, *Angew. Chem. Int. Ed.* **2007**, *46*, 3934-3937.
[4] S. Deller, S. Sollner, R. Trenker-El-Toukhy, I. Jelesarov, G. M. Gübitz, P. Macheroux, *Biochemistry* **2006**, *45*, 7083-7091.
[5] M. Hall, C. Stueckler, B. Hauer, R. Stuermer, T. Friedrich, M. Breuer, W. Kroutil, K. Faber, *Eur. J. Org. Chem*. **2008**, 1511-1516.
[6] A. Müller, B. Hauer, B. Rosche, *Biotechnol. Bioeng.* **2007**, *98*, 22-29.
[7] D. S. Blehert, B. G. Fox, G. H. Chambliss, *J. Bacteriol.* **1999**, *181*, 6254-6263.
[8] J. Buckman, S. M. Miller, *Biochemistry* **1998**, *37*, 14326-14336.
[9] J. F. Chaparro-Riggers, T. A. Rogers, E. Vazquez-Figueroa, K. M. Polizzi, A. S. Bommarius, *Adv. Synth. Catal.* **2007**, *349*, 1521-1531.
[10] A. Morokutti, A. Lyskowski, S. Sollner, E. Pointner, T. B. Fitzpatrick, C. Kratky, K. Gruber, P. Macheroux, *Biochemistry* **2005**, *44*, 13724-13733.
[11] C. Møller, M. S. Plesset, *Phys. Rev.* **1934**, *46*, 618-622.
[12] (a) T. H. Dunning, *J. Chem. Phys.* **1989**, *90*, 1007-1023; (b) R. A. Kendall, T. H. Dunning, R. J. Harrison, *J. Chem. Phys.* **1992**, *96*, 6796-6806.
[13] J. M. L. Martin, *Chem. Phys. Lett.* **1996**, *259*, 669-678.
[14] R. J. Bartlett, In *Coupled-Cluster Theory: An Overview of Recent Developments*; D. R. Yarkony, Ed.; *Advanced Series in Physical Chemistry: Modern Electronic Structure Theory*; World Scientific Publishing Co., Singapore, **1995**, Vol. *2*, pp. 1047-1131.
[15] S. K. Min, E. C. Lee, H. M. Lee, D. Y. Kim, D. Kim, K. S. Kim, *J. Comput. Chem.* **2008**, *29*, 1208-1221.
[16] a) C. J. Cramer, D. G. Truhlar, *Acc. Chem. Res.* **2008**, *41*, 760–768; b) A. V. Marenich, R. M. Olson, C. P. Kelly, C. J. Cramer, D. G. Truhlar, *J. Chem. Theory Comput*. **2007**, *3*, 2011–2033.
[17] a) Y. Zhao, D. G. Truhlar, *Theor. Chem. Acc.* **2008**, *120*, 215-241; b) Y. Zhao, D. G. Truhlar, *Acc. Chem. Res.* **2008**, *41*, 157-167.
[18] a) W. J. Hehre, R. Ditchfield, J. A. Pople, *J. Chem. Phys.* **1972**, *56*, 2257-2261; b) T. Clark, J. Chandrasekhar, G. W. Spitznagel, P. v. R. Schleyer, *J. Comp. Chem.* **1983**, *4,* 294-301; c) R. Krishnan, J. S. Binkley, R. Seeger, J. A. Pople, *J. Chem. Phys.* **1980**, *72*, 650-654; d) P. M. W. Gill, B. G. Johnson, J. A. Pople, M. J. Frisch, *Chem. Phys. Lett*. **1992**, *197*, 499-505;
[19] M. Valiev, E. J. Bylaska, N. Govind, K. Kowalski, T. P. Straatsma, H. J. J. Van Dam, D. Wang, J. Nieplocha, E. Apra, T. L. Windus, W. A. de Jong, *Comput. Phys. Commun.* **2010**, *181*, 1477-1489.

[20] a) M. W. Schmidt, K. K. Baldridge, J. A. Boatz, S. T. Elbert, M. S. Gordon, J. H. Jensen, S. Koseki, N. Matsunaga, K. A. Nguyen, S. Su, T. L. Windus, M. Dupuis, J. A. Montgomery, *J. Comput. Chem.* **1993**, *14*, 1347–1363; b) M. Higashi, A. V. Marenisch, R. M. Olson, A. C. Chamberlin, J. Pu, C. P. Kelly, J. D. Thompson, J. D. Xidos, J. Li, T. Zhu, G. D. Hawkins, Y. Y. Chuang, P. L. Fast, B. J. Lynch, D. A. Liotard, D. Rinaldi, J. Gao, C. J. Cramer, D. G. Thruhlar, *GAMESSPLUS - Version 2009*, University of Minnesota, Minneapolis, **2009**.

[21] G. Schaftenaar, J. H. Noordik, *J. Comput.-Aided Mol. Des.* **2000**, *14*, 123-134.

[22] H. Saimoto, K. Nishio, H. Yamamoto, M. Shinoda, *Bull. Chem. Soc. Jpn.* **1983**, *56*, 3093-3098.

[23] L. P. Christensen, *Photochemistry* **1999**, *51*, 969-974.

[24] J. Boukouvalas, R. P. Loach, *J. Org. Chem.* **2008**, *73*, 8109-8112.

[25] B. B. M. Ostermeier, C. Korff, G. Helmchen, *Eur. J. Org. Chem*. **2003***, 17,* 3453–3459.
